# Supplementary material for: Predicting the bacterial host range of plasmid genomes using the language model-based one-class support vector machine algorithm
Source: Microb Genom. 2025 Feb 11;11(2):001355. doi: 10.1099/mgen.0.001355 (PMC12282233; doi:10.1099/mgen.0.001355)
Supplement: Uncited Supplementary Material 1. [file mgen-11-01355-s001.pdf]

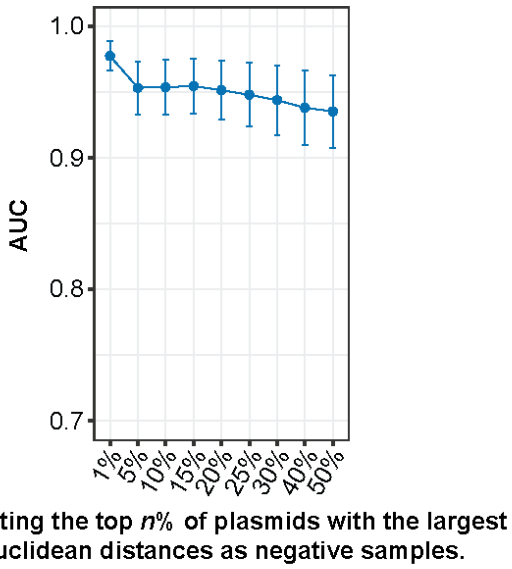

Figure S1. We ranked the non-positive plasmids by their Euclidean distances in descending order and then selected the top 1%, 5%, 10%, 15%, 20%, 25%, 30%, 40%, and 50% as negative samples. We calculated the *AUC* for different host taxonomic units at the genus level.

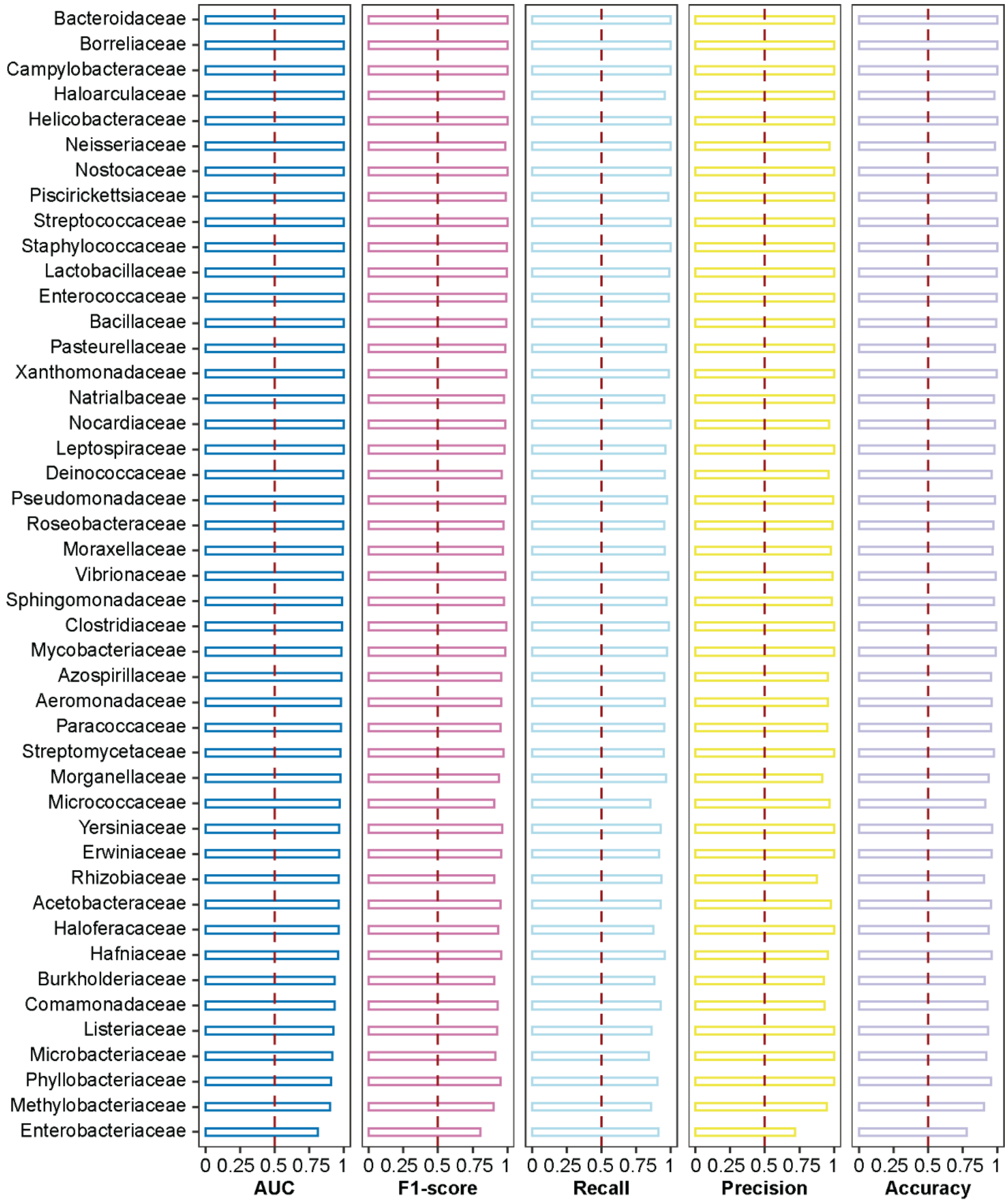

Figure S2. The evaluation results of model performance at the family level. The y-axis represents different families, while the x-axis represents various evaluation metrics, including *AUC*, *F1-score*, *Recall*, *Precision*, and *Accuracy*. The negative samples in the test set were generated based on sequence feature distances.

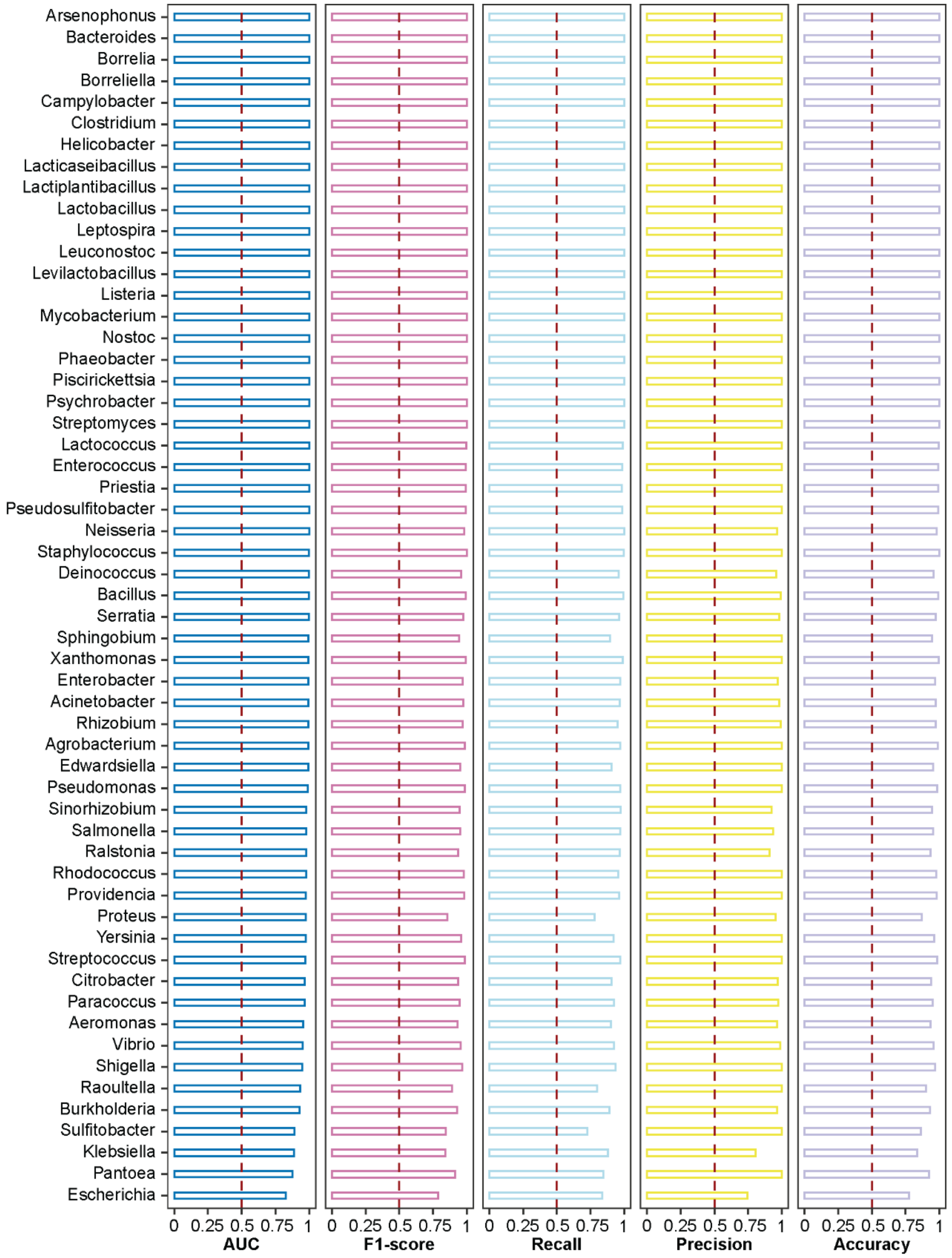

Figure S3. The evaluation results of model performance at the genus level. The y-axis represents different families, while the x-axis represents various evaluation metrics, including *AUC*, *F1-score*, *Recall*, *Precision*, and *Accuracy*. The negative samples in the test set were generated based on sequence feature distances.

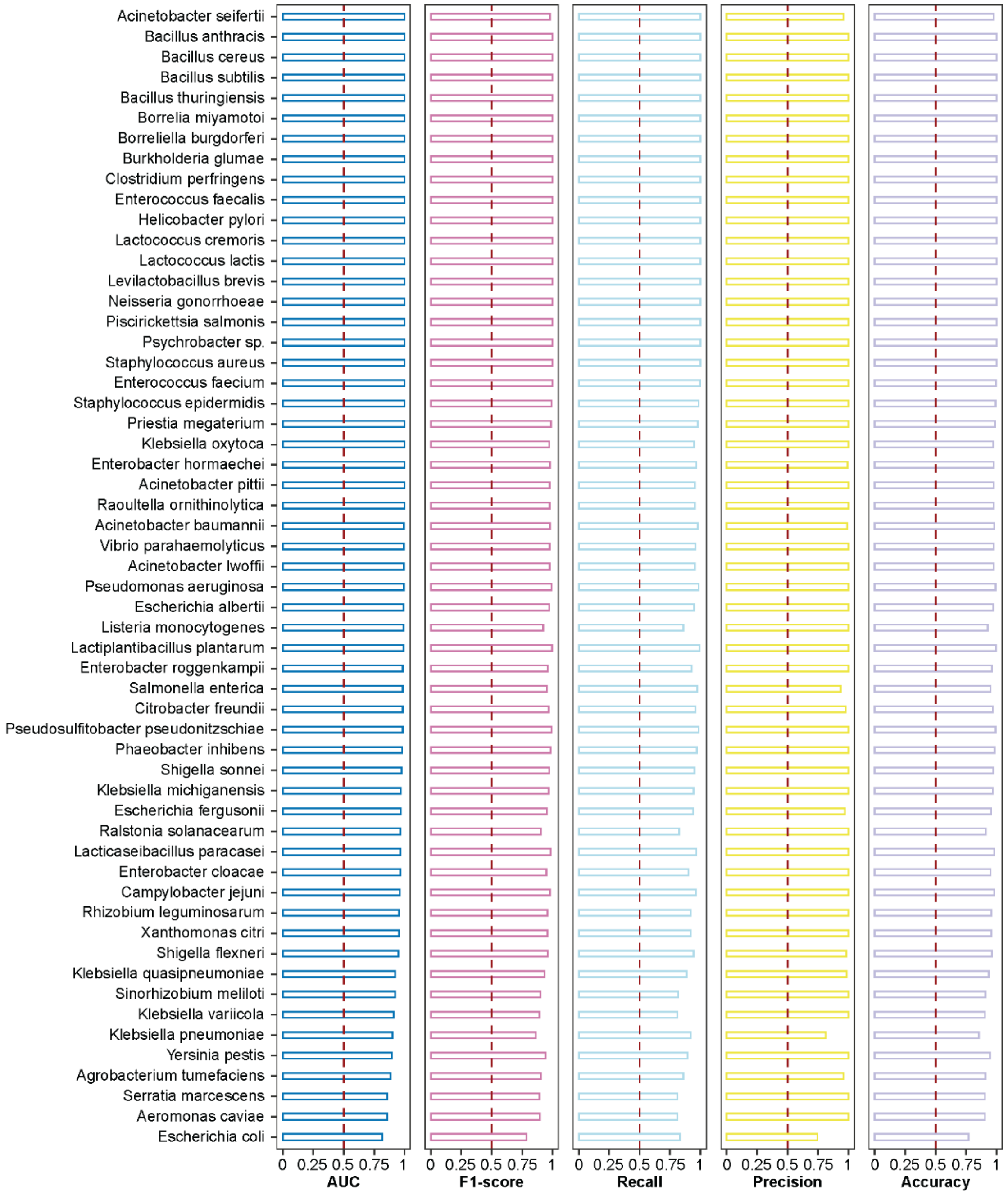

Figure S4. The evaluation results of model performance at the species level. The y-axis represents different families, while the x-axis represents various evaluation metrics, including *AUC*, *F1-score*, *Recall*, *Precision*, and *Accuracy*. The negative samples in the test set were generated based on sequence feature distances.

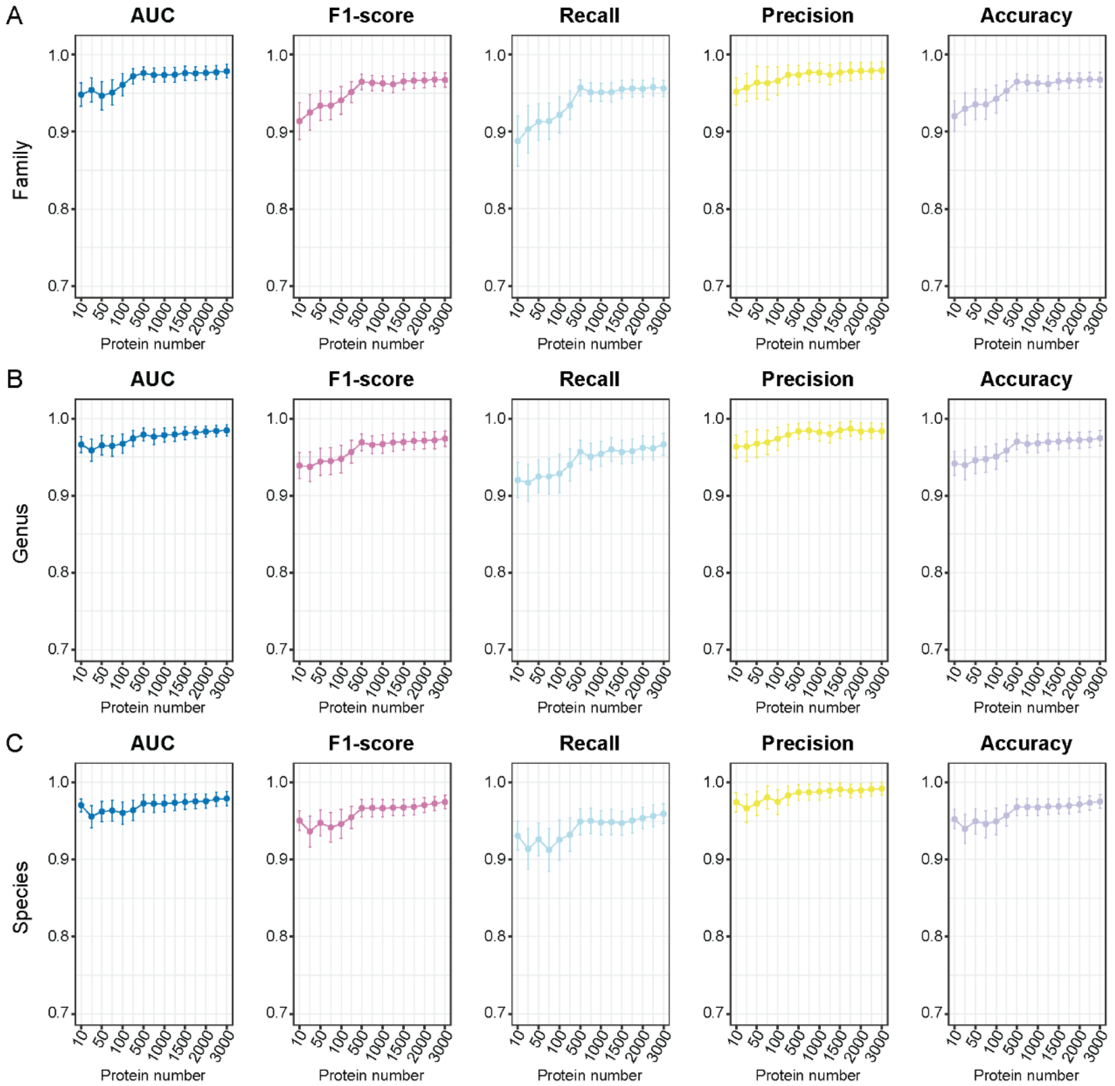

Figure S5. Performance evaluation results of models trained with different numbers of protein sequences at the taxonomic levels of family (A), genus (B), and species (C). The y-axis represents metrics including *AUC*, *F1-score*, *Recall*, *Precision*, and *Accuracy*. The x-axis indicates the number of randomly selected protein sequences used to construct the reference protein vector set, with values of 10, 25, 50, 75, 100, 300, 500, 800, 1000, 1200, 1500, 1800, 2000, 2500, and 3000. The generation of negative samples in these analyses is based on plasmid sequence signature distance.
